# Atypical Presentations at Risk for Diagnostic Errors in Internal Medicine: A Scoping Review

**DOI:** 10.1007/s11606-025-09901-z

**Published:** 2025-10-14

**Authors:** Yukinori Harada, Ren Kawamura, Masashi Yokose, Hardeep Singh, Taro Shimizu

**Affiliations:** 1 Department of Diagnostic and Generalist Medicine, Dokkyo Medical University, Mibu, Japan; 2 Center for Innovations in Quality, Effectiveness and Safety, Michael E. DeBakey Veterans Affairs Medical Center, Houston, TX USA; 3 Health Services Research Section, Department of Medicine, Baylor College of Medicine, Houston, TX USA

**Keywords:** atypical presentations, diagnostic errors, internal medicine

## Abstract

**Background:**

Little is known about how atypical disease presentations lead to diagnostic errors. Better definitions of atypical presentations may improve our understanding. We aimed to describe how atypical presentations were defined in studies of diagnostic errors in internal medicine.

**Methods:**

We included papers that described the association between atypical presentations in adult patients and diagnostic errors in internal medicine, indexed from database inception to July 31, 2025. We excluded case reports and conference abstracts. The data were extracted through MEDLINE, Web of Science, CINAHL, Embase, Cochrane Library, Google Scholar, and MedRxiv searches.

**Results:**

We included 56 papers in this review. Thirty studies included a definition of atypical presentation, but there was a considerable heterogeneity among the definitions. Using basic qualitative content analysis, we developed a new approach (Primary, Suggestive, Uncommon, and Chameleon features—the PSUC approach) to describe clinical presentations and identified four patterns at high risk of diagnostic errors. Pattern 1 lacks Primary disease features (i.e., features always written in textbooks) but has Suggestive features (i.e., stimulating consideration of specific disease); Pattern 2 lacks Primary features but Suggestive and Uncommon features (i.e., uncommon but known features in specific disease) are present. Pattern 3 lacks Primary and Suggestive features but has Uncommon features and Pattern 4 is similar to 3 but with Chameleon features (i.e., primary features for other diseases).

**Discussion:**

Atypical presentations in studies of diagnostic errors in internal medicine currently have high heterogeneity. A new approach to classify atypical presentations may be useful and warrants investigation in future research.

**Trial Registration:**

Open Science Framework www.osf.io/27d5m.

**Supplementary Information:**

The online version contains supplementary material available at 10.1007/s11606-025-09901-z.

## INTRODUCTION

Diagnostic errors, defined as “the failure to (a) establish an accurate and timely explanation of the patient’s health problem(s) or (b) communicate that explanation to the patient,”^1^ have emerged as a key concern in improving patient safety. Studies show the burden of diagnostic error to be considerable.^2–4^ While diagnostic errors can occur across care settings, internal medicine is one of the highest-risk care settings for diagnostic errors.^5,6^ Internal medicine physicians also frequently confront malpractice claims related to diagnostic errors.^7,8^ Since internal medicine covers a broad spectrum of complaints and diseases, diagnostic uncertainty can be much higher than in other specialties.^5,9^ Therefore, more complex and difficult diagnostic decisions are required in internal medicine, which can result in higher susceptibility to diagnostic errors.^5^

Diagnostic errors are usually related to multifactorial causes such as system-related errors, cognitive errors, and patient-related factors.^10–13^ Among these factors, patient-related factors can be present in almost all cases of diagnostic errors, and atypical presentations can contribute to many of these.^13^ Atypical presentations are described as “a shortage of prototypical features that are most frequently encountered in patients with the disease, features encountered in advanced presentations of the disease or simply features of the disease commonly listed in medical textbooks.”^14^ Atypical presentations can make diagnosis more challenging and distract from the diagnostic process. Recently, atypical presentations have been increasingly recognized for their impact on diagnostic errors. For example, atypical presentations were reported to be associated with a higher prevalence of diagnostic errors compared to typical presentations,^15–17^ and the major contributing factor to diagnostic errors.^13,18^ Moreover, previous studies suggested that if physicians were aware of some specific patterns of atypical presentations, the prevalence of diagnostic errors in patients with atypical presentations could be lower.^19,20^ These findings support the idea that developing strategies to increase awareness of the potential impact of atypical presentations and to prevent progression from an atypical presentation to diagnostic error seems to be a promising approach to improving patient safety.^13,17,21^

Atypical presentations seem to be especially an important issue for diagnostic errors in internal medicine: first, atypical presentations may be commonly observed (up to approximately 30%) in the internal medicine department;^16^ second, internal medicine physicians consider atypical presentations the most important contributor to diagnostic errors in their clinical practice;^22^ and third, atypical presentations can be an important contributing factor to a higher prevalence of diagnostic errors in internal medicine.^12,16^ However, knowledge gaps in this area persist, possibly due to the lack of consensus on the definition and measurement of atypical presentations and because a comprehensive conceptual model for understanding how atypical presentations progress to diagnostic errors is still lacking. Therefore, there is a need to systematically map and categorize how atypical presentations are defined in existing studies in order to support future research and clinical application.

This scoping review aimed to identify literature regarding the definitions of atypical presentations within the context of diagnostic errors in internal medicine. The primary review question was “What definitions and measurements have been used to identify atypical presentations in the studies investigating diagnostic errors in adult patients in internal medicine?”

Throughout this scoping review, the key terms such as diagnostic errors, atypical presentations, and internal medicine were defined as follows:

- Diagnostic errors—defined as the failure to (1) establish an accurate and timely explanation of the patient’s health problems or (2) communicate that explanation to the patient,^1^ and include delayed, wrong, and missed diagnosis.^12^
- Atypical presentations (working definition)—defined as patient demographics (e.g., age, sex, and race), symptoms and signs, test results, or clinical course, including the response to treatment, deviated from the prototypical patterns for the final diagnosis.
- Internal medicine—defined as a medical specialty concerned with the diagnosis and treatment of diseases of the internal organ systems of adults.

## METHODS

### Protocol and Registration

This scoping review was conducted following the Joanna Briggs Institute methodology for scoping reviews^23,24^ and in line with the Preferred Reporting Items for Systematic Reviews and Meta-Analyses Extension for Scoping Reviews.^25^ Our protocol was registered prospectively with the Open Science Framework on January 30, 2024 (https://osf.io/27d5m), and was also published on March 25, 2024, from elsewhere.^26^

### Eligibility Criteria

At the time of protocol development, the original eligibility criteria restricted studies that explored the association between atypical presentations and diagnostic errors (using any clear definition, criteria, or measurement to identify atypical presentations and diagnostic errors) in adult patients with internal medicine. However, after the full-text screening began, there was indication that only a few studies might meet this criterion. Therefore, we removed the requirement “using any clear definition or criteria or measurement to identify atypical presentations and diagnostic errors” from the criteria to make them less strict. Regarding the type of sources, quantitative, qualitative, and mixed-method studies, systematic reviews, and text and opinion papers were considered eligible. Papers were excluded if they investigated only either atypical presentations or diagnostic errors, focused on the pediatric population, focused on outside internal medicine, and were case reports, case series, or conference abstracts. Papers written in all languages were included.

### Information Sources

To identify potentially relevant documents, the following bibliographic databases were searched from inception to July 31, 2025: MEDLINE (PubMed), Science Citation Index Expanded (Web of Science), Embase, Cumulative Index to Nursing and Allied Health Literature, Google Scholar, Cochrane Library, and MedRxiv. The search strategies were drafted by an experienced librarian and further refined through team discussion. We performed the original search on February 2, 2024. An updated search was conducted on August 18, 2025, to capture new evidence. The original and updated search strategies for MEDLINE (PubMed) can be found in Appendix 1. The search results were exported into EndNote (Clarivate Analytics, Philadelphia, USA), and duplicates were removed. The reference lists of relevant papers were also used as additional sources.

### Selection of Sources of Evidence

Following the search, all identified records were collated and uploaded into Covidence (Covidence, Melbourne, Australia). Following a pilot test using the same 20 papers, two reviewers (YH and RK) independently evaluated the titles and abstracts. Subsequently, the reviewers independently evaluated the full text of potentially relevant papers. Any disagreements between the reviewers at each stage of the selection process were resolved through discussion. We used Google Translate and Google Lens to read papers written in languages other than English. During the entire screening process, the reviewers referenced the working definitions of atypical presentations. During the title and abstract screening, the reviewers intended to increase sensitivity not to miss any relevant studies; this means that they did not consider whether each study used any clear definition, criteria, or measurement to identify atypical presentations and diagnostic errors or not.

### Data Charting Process

The reviewers (YH and RK) jointly developed a data-charting form to determine which variables to extract. The reviewers independently charted data from each eligible paper. During the data charting process, the reviewers referenced the working definitions of atypical presentations. Any disagreements were resolved through discussion between the reviewers.

### Data Items

We abstracted data on paper characteristics, target population, total number of participants, participant characteristics, the definitions and measurements to identify atypical presentations, the definition and measurements to identify diagnostic errors, the description or data about the association between atypical presentations and diagnostic errors, and the factors or characteristics that are related to atypical presentations.

### Synthesis of Results

First, we summarized how the association between atypical presentations and diagnostic errors was investigated and described across the included papers. Second, we conducted a basic qualitative content analysis to identify the key components of atypical presentations and classify the various types, judging from the papers that described the definitions or measurements to identify atypical presentations. According to the guidelines outlined in the Joanna Briggs Institute’s scoping review methodology,^27^ we followed Elo and Kyngäs’ qualitative inductive content analysis process,^28^ as well as other methodological references.^29,30^ In detail, (1) all authors agreed to define the definition of atypical presentations as a unit of analysis and to analyze only the texts describing the definition of atypical presentations extracted from each article by the data extraction form. (2) YH and RK familiarized themselves with the data during full-text screening and extraction. (3) YH conducted open coding, created tentative descriptive categories, and abstracted the meanings in each category. (4) The categories were revised several times by discussion between YH, RK, and MY until they reached a consensus on seven categories, including a shortage of prototypical features and/or presence of features with unexpected values, absence of classical symptoms but the presence of symptoms and signs suggesting the target disease, presence of less common symptoms of the disease without classical symptoms, presence of non-classical symptoms that are classical for other diseases, and subjective judgment by physicians. (5) YH and RK looked for the underlying meanings running through these descriptive categories, interpreted the latent content, and formulated the theme that “atypical presentations are described by the combination of presence or absence of the four types of features.” YH and RK also defined each feature type with examples. (6) According to the theme and the four types of features, YH and RK sorted all definitions of atypical presentations into six new categories and developed a coding scheme. All the authors agreed with the coding scheme and named the four types of features as primary features, suggestive features, uncommon features, and chameleon features. (7) MY tested the coding scheme, and YH, RK, and MY revised the categories by solving coding discrepancies. Finally, we developed the five categories: the four categories that describe patterns of atypical presentations and the one category that describes how atypical presentations are subjectively measured.

## RESULTS

### Selection of Sources of Evidence

**Figure 1:**
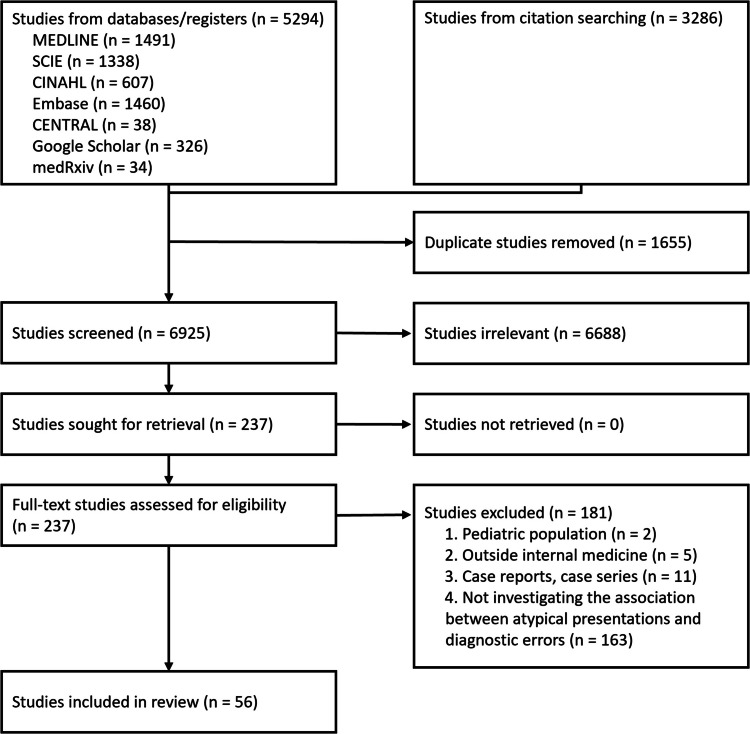
Flow diagram of the screening.

After duplicates were removed, 6925 citations were identified from searches of electronic databases and review article references. Based on the title and the abstract, 6688 were excluded, and 237 full-text articles (including 28 articles written in other than English: 9 in Chinese, 5 in Russian, 3 in German, 2 in French, 2 in Korean, 2 in Polish, 1 in Bulgarian, 1 in Hungarian, 1 in Italian, 1 in Japanese, and 1 in Spanish) were retrieved and assessed for eligibility. Of these, 181 were excluded for the following reasons: 2 were exclusive to the pediatric population, 5 focused outside internal medicine, 11 were case reports or case series, and 163 did not investigate the association between atypical presentations and diagnostic errors. The remaining 56 studies were considered eligible for this review (Fig. 1).^14,16–18,20–22,31–79^

### Characteristics of Sources of Evidence

Six studies were written in languages other than English: 2 in Chinese,^54,73^ 2 in Russian,^39,62^ 1 in Hungarian,^57^ and 1 in Korean.^63^ Most of the included sources of evidence were observational studies or cross-sectional studies (*n* = 40)^16,20,22,31,32,34–38,40–42,44–51,53–58,60–63,65–68,72–76^ and other types of studies included systematic or narrative reviews (*n* = 5),^14,17,18,33,39^ qualitative research (*n* = 5),^21,43,64,69,77^ questionnaire survey (*n* = 2),^70,71^ experimental study (*n* = 2),^78,79^ commissioned report (*n* = 1),^52^ and dissertation (*n* = 1).^59^ A total of 16 studies were disease agnostic.^14,16–18,21,22,41,43,46,52,59–61,69,76,79^ Among 40 studies that focused on specific diseases,^20,31–40,42,44,45,47–51,53–58,62–68,70–75,77,78^ 32 studies focused on acute diseases,^20,31–34,37–39,42,44,45,47,50,51,53–58,62–68,72–75,78^ and vascular diseases were the most common target disease (*n* = 21).^20,32,33,36–39,42,45,47,53–57,62,67,68,72,73,78^ Regarding clinical settings, 33 studies focused on outpatients, including emergency departments,^14,16,17,20,22,31,32,35,38,41–47,51,53,54,56,58,59,61–65,67,69,72,73,76,78^ 2 studies focused on inpatients including intensive care unit,^50,68^ 3 studies focused on both outpatients and inpatients,^37,55,66^ and the remaining 18 studies were not obvious.^18,20,21,33,34,39,40,48,49,52,57,60,70,71,74,75,77,79^

### Results of Individual Sources of Evidence

The results of individual sources of evidence are shown in Table 1. One study described that there was no effect of atypical presentations on diagnostic errors;^37^ however, other studies described some kind of association between atypical presentations and diagnostic errors as follows: (1) atypical presentations were more frequently observed in patients with diagnostic errors (compared to patients without diagnostic errors), diagnostic errors occurred more frequently in patients with atypical presentations (compared to patients with typical presentations), or atypical presentations were associated with the increased risk of diagnostic errors (*n* = 31);^16–18,20,31–33,36,38,39,42,44,46,50,51,53,55–58,61–63,65–68,73,74,76,78^ (2) atypical presentations were considered a contributing factor to diagnostic errors (*n* = 14);^14,21,22,41,43,52,59,60,64,69,70,75,77,79^ (3) time to diagnosis was longer in patients with atypical presentations (compared to patients with typical presentations) (*n* = 9);^34,35,37,45,47–49,71,72^ and (4) atypicality was greater in patients with diagnostic errors (compared to patients without diagnostic errors) (*n* = 1).^54^

**Table 1:** Results of Individual Sources of Evidence

Author and year of publication	Country	Article type	Number of participants	Age	Sex	Target disease	Definitions of atypical presentation	Association between atypical presentations and diagnostic errors
Abbott 1990^31^	USA	Retrospective study	65	Prompt diagnosis group: mean, 25.9 Delayed diagnosis group: mean, 25.6	Women, 100%	Ectopic pregnancy	N/A	Atypical or absent pain was observed in 92% of patients in the delayed diagnosis group
Al-Mallah 2010^32^	Bahrain, Kuwait, Oman, Qatar, UAE, Yemen	Prospective study	3197	Mean, 54	Women, 14%	ST-segment elevation myocardial infarction	N/A	Patients with delayed presentation had a higher prevalence of atypical symptoms
AWARE-IBD Diagnostic Delay Working Group 2024^77^	UK	Qualitative research	20	N/A	Women, 65.0%	Inflammatory bowel disease	Atypical presentations were defined as few or no prototypical features or unexpected test values	Atypical presentations were a possible factor contributing to diagnostic delay
Bakradze 2018^33^	USA	Narrative review	N/A	N/A	N/A	Stroke	N/A	Patients with non-traditional symptoms were more likely to be missed in the emergency department than patients who presented with traditional stroke symptoms
Bewersdorf 2019^34^	Germany	Retrospective study	18	Mean, 54.7	Women, 50%	Herpes simplex virus encephalitis	N/A	Patients with normocellular cerebrospinal fluid were associated with a delay in the establishment of the diagnosis in comparison to those with elevated cerebrospinal fluid cell counts on admission
Bjerager 2006^35^	Denmark	Retrospective study	84	Median, 66	Women, 35.7%	Lung cancer	Atypical presentations were defined as patients presenting with atypical symptoms that were not lung-related	Patients with atypical presentations experienced a longer diagnostic delay compared to patients with typical presentations
Bondarev 2024^75^	Republic of Moldova	Cross sectional study	152	N/A	N/A	Mechanical injury	N/A	Atypical symptoms were the second most common cause of diagnostic discrepancies between clinical and medico-legal diagnoses in brain injury
Brandler 2015^36^	USA	Retrospective study	72,984	Mean, 47.6	Women, 55%	Stroke	N/A	Patients who presented with atypical presentations were more likely to be missed by emergency medical service providers
Brieger 2004^37^	Argentina, Australia, Austria, Belgium, Brazil, Canada, France, Germany, Italy, New Zealand, Poland, Spain, UK, USA	Prospective study	20,881	Atypical presentation group: median, 72.9 Typical presentation group: median, 65.8	Atypical presentation group: women, 42.0% Typical presentation group: women, 32.5%	Acute coronary syndrome	Atypical presentations were defined as patients without chest pain	Patients with atypical presentations took longer to present to the hospital and were more likely misdiagnosed than those with typical presentations
Canto 2000^38^	USA	Prospective study	434,877	Atypical presentation group: mean, 74.2 Typical presentation group: mean, 66.9	Atypical presentation group: women, 49.0% Typical presentation group: women, 38.0%	Myocardial infarction	Atypical presentations were defined as patients without chest pain	Patients with atypical presentations were significantly less likely to be admitted with an initial diagnosis of myocardial infarction
Demin 2019^39^ (Written in Russian)	Russia	Narrative review	N/A	N/A	N/A	Stroke	Atypical presentations were defined as symptoms not resembling a stroke and "similar to other (non-vascular) events."	Atypical presentations increase the likelihood of misdiagnosis
Eckardt 1997^40^	Germany	Prospective study	87	Mean, 43.7	Women, 39%	Achalasia	N/A	The duration of symptoms to diagnosis was similar in patients with and without atypical symptoms
Ely 2012^41^	USA	Cross-sectional study	202	Mean, 51.8	Women, 38.5%	Disease agnostic	N/A	One common lesson physicians learned after diagnostic errors was to be wary of atypical disease presentations
Fang 2022^42^	China	Retrospective study	202	Atypical presentation group: mean, 58.1 Typical presentation group: mean, 55.9	Atypical presentation group: women, 37.62% Typical presentation group: women, 40.59%	Acute coronary syndrome	Atypical presentations were defined as patients with acute coronary syndrome whose symptoms manifested primarily as throat discomfort	The rate of misdiagnosis was 56.82% in patients with atypical presentations
Goyder 2015^43^	UK	Qualitative research	36	N/A	Women, 33%	Disease agnostic	Atypical presentations were defined as patients with symptoms that did not fit a known and recognizable pattern	Diagnostic errors can occur when patients present with atypical presentations. For example, one general practitioner described missing an opportunity to diagnose an abdominal aortic aneurysm
Graff 2000^44^	USA	Retrospective study	2144	N/A	N/A	Acute appendicitis	N/A	Patients whose diagnosis was missed by the physician had significantly higher atypical symptoms and signs
Grosmaitre 2013^45^	France	Retrospective study	255	Mean, 84.6	Women, 62.7%	ST-segment elevation myocardial infarction	Atypical presentations were defined as patients presented without chest pain	Patients with atypical presentations experienced longer prehospital delays
Harada 2023^18^	Japan	Systematic review	563	Mean, 42.0	Women, 46.9%	Disease agnostic	Atypical presentations were categorized based on descriptions in the articles and the physician-researcher judgment	In total, 39.8% of cases were categorized as having atypical presentations among the case reports describing diagnostic errors. Atypical presentations were the most important factor contributing to diagnostic errors
Harada 2023^16^	Japan	Retrospective study	930	Mean, 55.7	Women, 49.2%	Disease agnostic	Atypical presentations were assessed based on item 12 of the revised Safer Dx Instrument	Diagnostic errors were significantly higher in the group of atypical presentation than in the group of typical presentation
Hautz 2019^46^	Switzerland	Prospective study	755	Mean, 65.1	Women, 42.7%	Disease agnostic	Atypical presentations were measured by asking diagnosing physicians to rate how typical they deemed the patient’s presentation for the assigned diagnosis on a customized Likert scale questionnaire	When physicians rated the presentation as atypical, it predicted later diagnostic discrepancies well
Hsieh 2011^47^	Taiwan	Retrospective study	163	Mean, 58	Women, 21%	Acute aortic dissection	Atypical presentations were defined as those free from typical painful sensations and included syncope without preceding pain, neurological deficits, shortness of breath, nonspecific abdominal or chest discomfort, and other common atypical presentations	The time spent from hospital arrival to diagnosis was significantly longer in those with atypical presentations than typical presentations, and delay in diagnosis was observed more frequently in the group with atypical presentations
Indelicato 2020^48^	European countries	Retrospective study	611	Median, 31	Women 47%	Friedreich’s ataxia	Atypical presentations were defined as patients with non-neurological onset	The time to diagnosis in the group of atypical presentations was significantly longer compared to the group of typical presentations
Kaufmann 2018^49^	Switzerland	Retrospective study	1059	Median, 47	Women, 73%	Multiple sclerosis	Atypical presentations were defined as the remainder of common symptoms	Atypical presentations were associated with an extended time to diagnosis
Kollef 1991^50^	USA	Retrospective study	28	Misdiagnosis group: mean, 51.8 Correct diagnosis group: mean, 50.1	Misdiagnosis group: women, 55.6% Correct diagnosis group: women, 66.7%	Pneumothorax	Atypical presentations were defined as atypical locations of pneumothorax	Atypical presentations were more frequently observed in patients with diagnostic errors
Komagamine 2022^51^	Japan	Retrospective and prospective study	285	Median, 82	Women, 65.3%	Urinary tract infection	Atypical presentations were defined as the absence of any new urinary tract symptoms and signs	Patients with atypical presentations were less likely to be correctly diagnosed with urinary tract infections
Kostopoulou 2008^52^	UK	Commissioned report	N/A	N/A	N/A	Disease agnostic	Atypical presentations were defined as a shortage of typical cues or may include cues with unexpected values	Atypical presentations were identified as the features of diagnostic difficulty
Kostopoulou 2008^14^	UK	Systematic review	N/A	N/A	N/A	Disease agnostic	Atypical presentations were defined as patients have a shortage of prototypical features and may also have features with unexpected values	Atypical presentations were identified as one of the potential diagnostic difficulties
Lever 2013^53^	USA	Retrospective study	189	Mean, 70.4	Women, 49.7%	Ischemic stroke	Atypical presentations were defined as nontraditional symptoms	Patients with atypical presentations were misdiagnosed more than patients with typical presentations. Among the patients whose diagnosis of stroke was missed, 79.3% had atypical presentations
Liang 2009^54^ (Written in Chinese)	China	Retrospective study	63	Mean, 51.3	Women, 41.3%	Pulmonary embolism	Atypical presentations were defined as patients with low Wells and modified Geneva scores	The grades of atypical presentations were significantly greater in the diagnostic errors group than in the correct diagnosis group
Liberman 2020^55^	USA	Retrospective study	478	N/A	N/A	Ischemic stroke	Atypical presentations were defined as non-traditional stroke symptoms	Among diagnostic error cases, 35.7% involved exclusively atypical presentations
Liberman 2025^78^	USA	Experimental study	27	N/A	N/A	Acute stroke	Atypical presentations were defined as acute ischemic stroke with mild symptoms	35% of physicians misdiagnosed an atypical acute ischemic stroke case, whereas 0% of them misdiagnosed a typical acute ischemic stroke case
Maharaj 2012^56^	South Africa	Retrospective study	161	Median, 54	Women, 33.5%	Acute myocardial infarction	Atypical presentations were defined as patients not consistent with typical presentations	Among cases where the reason for the diagnostic delay was recorded, 12.9% of patients presented with atypical symptoms
Márk 1994^57^ (Written in Hungarian)	Hungary	Retrospective study	97	Misdiagnosis group: mean, 72.0 Correct diagnosis group: mean, 67.3	Misdiagnosis group: women, 35% Correct diagnosis group: women, 48%	Acute myocardial infarction	Atypical presentations were defined as when chest pain did not occur, but weakness, fatigue, suffocation, sweating and left ventricular failure did	Atypical presentations were observed in 50% and 15% in patients with and without diagnostic errors, respectively
Martin 2023^58^	France	Retrospective study	119	Mean, 71	Women, 49.6%	Acute mesenteric ischemia	N/A	Atypical presentations were observed in 28% of patients with a delayed diagnosis
Matin 2022^59^	USA	Dissertation	N/A	N/A	N/A	Disease agnostic	N/A	Atypical presentation was one of the most frequently contributing factors to diagnostic errors
Matulis 2020^22^	USA	Cross-sectional study	65	N/A	Women, 44%	Disease agnostic	N/A	Atypical presentations were the most frequently cited individual-level factors contributing to diagnostic errors
Middleton 1989^60^	USA	Retrospective study	142	N/A	N/A	Disease agnostic	N/A	Atypical presentation was the cause of diagnostic errors in 17% of those aged 64 years or older and 10% in those aged 65 years or younger
Mutlak 2025^79^	UK	Experimental study	65	Mean, 24.3	Women, 58.0%	Disease agonistic	Atypical presentations were defined as symptoms or signs that deviate from textbook cases	Recognition of non-classical presentations at baseline was low, and 58.5% (38/65) of students acknowledged difficulty in recognizing atypical symptoms of commonly misdiagnosed conditions. Atypical presentations represent the main categories of error contributing to misdiagnosis in the study cohort
Newman-Toker 2022^17^	USA	Systematic review	N/A	N/A	N/A	Disease agnostic	N/A	Atypical presentations were the strongest and most consistent predictors of increased risk for missed diagnoses across diseases studied
Okafor 2016^61^	USA	Retrospective study	209	N/A	N/A	Disease agnostic	N/A	Atypical presentations were involved in 31.1% of diagnostic errors
Pankin 2001^62^ (Written in Russian)	Russia	Retrospective study	1500	N/A	N/A	Myocardial infarction	Atypical presentations were defined as patients not consistent with the typical presentations that were defined as severe and prolonged anginal attacks that cannot be controlled by nitroglycerin	Late diagnosis, overdiagnosis, and underdiagnosis were significantly more frequent in patients with atypical presentations than patients with typical presentations
Park 2020^63^ (Written in Korean)	Korea	Retrospective study	77	Delayed diagnosis group: mean, 68.4 Timely diagnosis group: mean, 65.8	Delayed diagnosis group: women, 64.7% Timely diagnosis group: women, 62.8%	Acute angle-closure glaucoma	Atypical presentations were defined as patients who mainly complained of extraocular symptoms	The prevalence of atypical presentations was higher in the delayed diagnosis group than in the timely diagnosed group
Peng 2016^64^	Taiwan	Qualitative research	20	Mean, 46	Women, 5%	Hollow organ perforation	N/A	Atypical presentations were one of the four formulated themes for delayed, wrong or missed diagnoses. Atypical presentations comprised 26% of the reasons for inaccurate or delayed diagnosis of hollow organ perforation
Rothrock 1995^65^	USA	Retrospective study	174	Misdiagnosis group: mean, 28.5 Correct diagnosis group: mean, 27.6	Women, 100%	Appendicitis	N/A	Atypical presentations were more frequent in misdiagnosed cases
Saraswat 2019^66^	India	Retrospective study	18	Mean, 44.7	Women, 39%	Leprosy	Atypical presentations were defined as patients of leprosy who lacked classical presentation of leprosy	All cases of atypical presentations were misdiagnosed at the time of initial presentations
Schiff 2022^21^	USA	Qualitative research	836	N/A	N/A	Disease agnostic	N/A	Atypical presentations were one of the 5 leading types of generic pitfalls
Schrock 2012^67^	USA	Retrospective study	429	Discordant diagnosis group: median, 57 Concordant diagnosis group: median, 60	Discordant diagnosis group: women, 63% Concordant diagnosis group: women, 62%	Transient ischemic attack	Atypical presentations were defined as patients with headache, tingling, involuntary movement, flashing lights, wavy lines, dizziness, confusion, or incontinence	Atypical presentations were associated with a discordant diagnosis of transient ischemic attack
Shiyovich 2010^68^	Israel	Retrospective study	268	Incorrect diagnosis group: mean, 70.5 Correct diagnosis group: mean, 74.2	Incorrect diagnosis group: women, 60% Correct diagnosis group: women, 56%	Atrial flutter, atrial fibrillation	Atypical presentations were defined as patients with atrial flutter showing atypical ƒ waves upright in leads II, III, and aVF and inverted in lead V1	The misdiagnosis rate was higher in the group of atypical presentations
Su 2017^69^	Taiwan	Qualitative research	10	Mean, 41.3	Women, 10%	Disease agnostic	N/A	As a cause of diagnostic errors, atypical presentations accounted for 20%
Suneja 2022^70^	USA, Canada, Puerto Rico	Questionnaire survey	533	N/A	N/A	Tuberculosis, Non-tuberculous mycobacteria, syphilis, epidural abscess, infective endocarditis, endemic fungal infections	N/A	Among the factors contributing to diagnostic errors, atypical presentations accounted for 8% of tuberculosis, 7% of Non-tuberculous mycobacteria, 9% of syphilis, 10% of epidural abscess, 15% of infective endocarditis, and 3% of endemic fungal infections
Tan 2021^71^	the Netherlands	Questionnaire survey	211	Median, 4.8	Women, 68.2%	Celiac disease	Atypical presentations were defined as patients without classical symptoms	Patients with atypical presentations were diagnosed at a significantly older age than those with typical presentations
Tolenaar 2013^72^	Italy, Canada, USA, Japan, Spain, Netherlands, Germany	Retrospective study	1162	Atypical presentation group: mean, 69.2 Typical presentation group: mean, 63.3	Atypical presentation group: women, 31.9% Typical presentation group: women, 33.1%	Type B acute aortic dissection	Atypical presentations were defined as patients presenting without any pain symptoms	Patients with atypical presentations had a longer mean time from admission to diagnosis than those with typical presentations
Watanabe 2025^76^	Japan	Retrospective study	321	Mean 59	Women, 46.4%	Disease agonistic	Atypical presentations were judged by the case reviewers	Atypical presentations were observed in 64.1% and 29.4% in patients with and without diagnostic errors, respectively
Wei 1995^73^ (Written in Chinese)	Taiwan	Retrospective study	548	N/A	Women, 48.0%	Cerebrovascular diseases	N/A	Of the misdiagnosis cases due to lack of knowledge, 76% were atypical presentations
Wilson 1988^74^	UK	Retrospective study	126	Missed diagnosis group: mean, 64 Correct diagnosis group: mean, 62	Missed diagnosis group: women, 49.2% Correct diagnosis group: women, 47.9%	Acute pancreatitis	N/A	68% of undiagnosed patients had presented atypically with known or suspected medical disease
Zarling 1983^20^	USA	Retrospective study	100	Missed diagnosis group: mean, 64.2 Correct diagnosis group: mean, 67.9	Missed diagnosis group: women, 46.8% Correct diagnosis group: women, 37.7%	Acute myocardial infarction	Atypical presentations were defined as symptoms other than the classic retrosternal chest pain that suggests a cardiac problem	Patients with atypical presentations were significantly less likely to be diagnosed before death than patients with typical presentations

*UAE*, United Arab Emirates; *USA*, United States of America; *UK*, United Kingdom

### Synthesis of Results

We detected the description of the definitions or measurements to identify atypical presentations in 34 papers (60.7%),^14,16,18,20,35,37–39,42,43,45–57,62,63,66–68,71,72,76–79^ among which 27 papers met the original eligibility criteria (79.4%).^14,16,18,20,35,39,42,45–55,57,63,66–68,71,76–78^ The definitions of atypical presentations varied among the studies, regardless of disease-agnostic or disease-specific studies. Even among the studies investigating the same diseases, atypical presentations were defined differently (e.g., acute coronary syndrome).^20,37,38,42,45,56,57,62^

**Figure 2:**
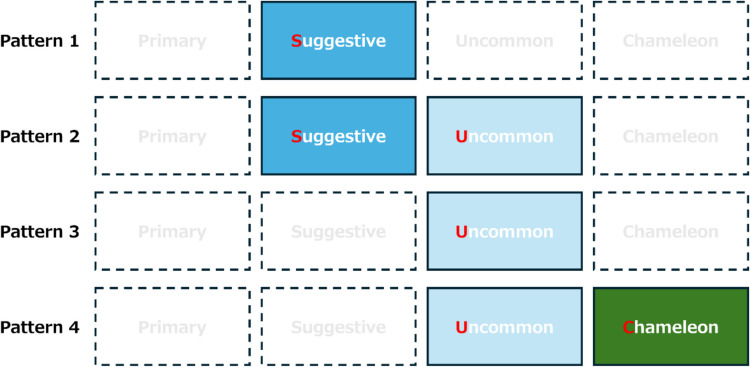
Four identified patterns of atypical presentations that related to diagnostic errors. The four patterns are described using a new approach to describe clinical presentation (Primary, Suggestive, Uncommon, and Chameleon features—the PSUC approach): Primary features—the core, traditional, classical features of the specific disease that are “always” written in the textbooks such as chest pain in myocardial infarction; Suggestive features—the features that are not primary features but can stimulate physicians to consider specific disease, for instance presence of shock and acute pulmonary edema makes one consider myocardial infarction; Uncommon features—those known to be one of the features in the specific disease, but their frequency is low, for instance presence of fatigue, balance problems, and dizziness/vertigo in multiple sclerosis: and Chameleon features—features that are not only not classical for the target disease but also known to be primary features for other diseases such as acute change in mental status in stroke. Pattern 1. Only Suggestive features present (e.g., isolated acute pulmonary edema in acute coronary syndrome); Pattern 2. Suggestive and Uncommon features are present, and two other features are absent (e.g., already known chronic obstructive pulmonary disease with new neurological symptoms in lung cancer); Pattern 3. Only Uncommon features present (e.g., isolated throat discomfort in acute coronary syndrome); and Pattern 4. Both Uncommon and Chameleon features are present, and the other two are absent (e.g., isolated psychiatric symptoms in acute stroke)

**Figure 3:**
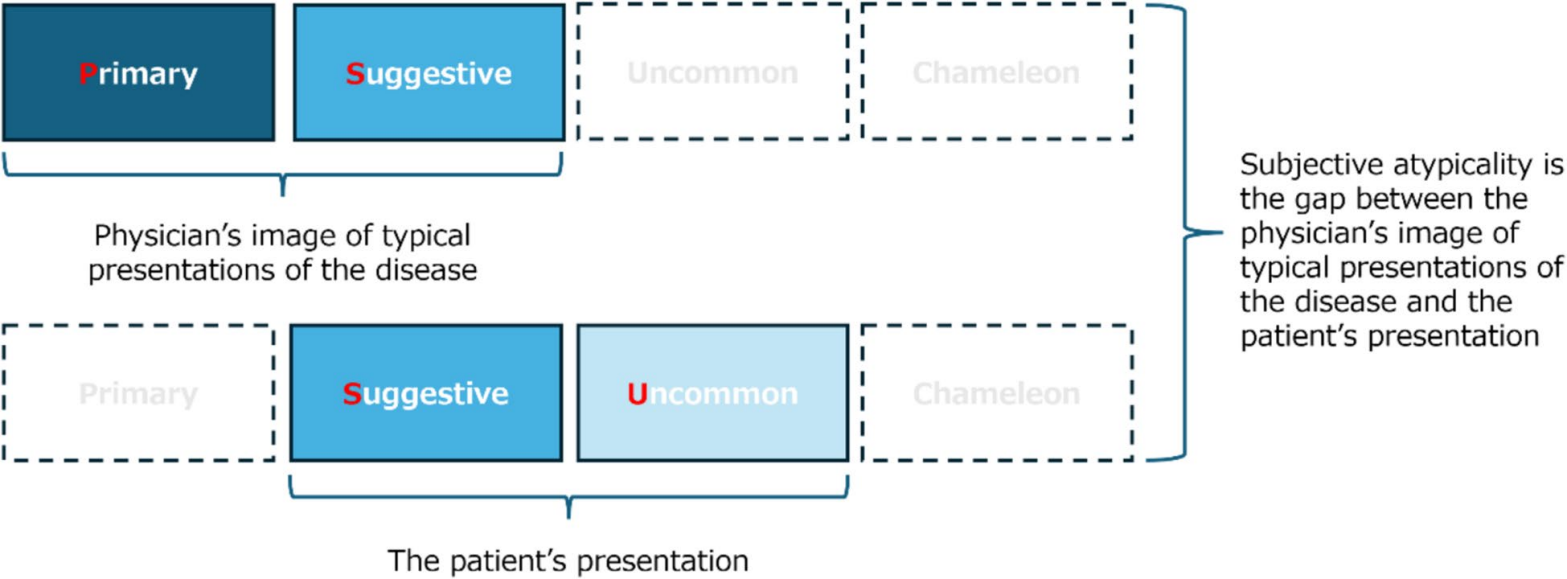
A schema to explain how atypicality can be measured. This schema shows that atypicality is measured by the gap between the physician’s image of typical presentations of the disease and the patient’s presentation. The schema uses a new approach to describe clinical presentation (Primary, Suggestive, Uncommon, and Chameleon features—the PSUC approach): Primary features—the core, traditional, classical features of the specific disease that are “always” written in the textbooks; Suggestive features—the features that are not primary features but can stimulate physicians to consider specific disease; Uncommon features—those known to be one of the features in the specific disease, but their frequency is low: and Chameleon features—features that are not only not classical for the target disease but also known to be primary features for other diseases

Using basic qualitative content analysis of the existing literature, we identified four key attributes to describe clinical presentations (PSUC approach): (1) Primary features—the core, traditional, classical features of the specific disease that are “always” written in the textbooks such as chest pain in myocardial infarction; (2) Suggestive features—the features that are not primary features but can stimulate physicians to consider a specific disease, for instance, the presence of shock and acute pulmonary edema makes one consider myocardial infarction; (3) Uncommon features—those known to be one of the features in the specific disease, but their frequency is low, for instance, the presence of fatigue, balance problems, and dizziness/vertigo in multiple sclerosis; and (4) Chameleon features—features that are not only not classical for the target disease but also known to be primary features for other diseases such as acute change in mental status in stroke. Using the combination of the presence or absence of these four components, we classified the definitions or measurements to identify atypical presentations described in the 34 papers into four patterns (Fig. 2): (1) Pattern 1. Only Suggestive features present (*n* = 5);^20,47,48,57,78^ (2) Pattern 2. Suggestive and Uncommon features are present, and two other features are absent (*n* = 15);^14,35,37,38,45,52,53,55,56,63,66,71,72,77,79^ (3) Pattern 3. Only Uncommon features present (*n* = 8);^42,49–51,54,62,67,68^ and (4) Pattern 4. Both Uncommon and Chameleon features are present, and the other two are absent (*n* = 1).^39^ In addition, using the model and insights from the several studies that were disease agnostic,^16,18,43,46,76^ we found that atypicality of presentations could be measured by the gap between the physician’s image of typical presentations of the disease and the patient’s presentation (Fig. 3).

## DISCUSSION

### Summary of Evidence

In this scoping review, we identified 56 primary studies addressing the association between atypical presentations and diagnostic errors in internal medicine. We found that there is still a paucity of research in patients with acute diseases other than cerebrovascular diseases and chronic diseases. We also found less than two-thirds (60.7%) of studies described the definitions or measurements to identify atypical presentations, and even among these studies, definitions or measurements to identify atypical presentations were heterogeneous. These findings support the idea that there is a need to develop universal definitions to identify and measure atypical presentations that are at risk of diagnostic errors.

### Limitations

Our scoping review has several limitations. First, during the study selection and data charting, we used a working definition of atypical presentations. Considering that the primary aim of this scoping review was to identify and synthesize how atypical presentations were defined and measured in previous studies, this approach could have influenced the inductive process in this scoping review and affected the study selection and data abstraction processes. However, it was not possible to screen studies and abstract data without any shared mental model about what kinds of descriptions refer to atypical presentations. Moreover, our working definition of atypical presentations intended that our scoping review considered not only presenting symptoms but also patient demographics, signs, test results, and clinical course as the components of atypical presentations, which maximized inclusion of any atypical presentations and helped preserve the inductive integrity of our scoping review as much as possible. Therefore, the effect of the working definition of atypical presentations on the results of this scoping review could be minimized. Second, although there was a working definition of atypical presentations, the lack of a standardized definition of atypical presentations reduced the clarity of the study selection and data abstraction processes, which might impact the reproducibility and interpretability of the review. Third, because this scoping review did not include studies outside of internal medicine, findings may not be generalizable to other specialties. Fourth, we could have overlooked some papers related to our scope, particularly papers that were not published or were not published within the databases that were searched, which potentially brings some bias to our understanding and conclusions.

### Implications

Despite most previous studies focusing on a few specific acute diseases, included studies support the association between atypical presentations and diagnostic errors irrespective of clinical settings and diseases. Moreover, we found that the association between atypical presentations and diagnostic errors was described within the following four types of categories: (1) atypical presentations were more frequently observed in patients with diagnostic errors (compared to patients without diagnostic errors), diagnostic errors occurred more frequently in patients with atypical presentations (compared to patients with typical presentations), or atypical presentations were associated with the increased risk of diagnostic errors; (2) atypical presentations were considered a contributing factor to diagnostic errors; (3) time to diagnosis was longer in patients with atypical presentations (compared to patients with typical presentations); and (4) atypicality was greater in patients with diagnostic errors. These categories may help researchers set the outcome of the study investigating the association between atypical presentations and diagnostic errors.

We found tremendous heterogeneity in the definitions and measurements of atypical presentations. As individual evidence in our scoping review showed, the heterogeneity of definition and measurements of atypical presentations among the studies is reported even in the same disease,^80^ which may prevent the systematic synthesis of available evidence and studies to address atypical presentations and diagnostic errors. As the basis of the standardized definition of atypical presentations that related to diagnostic errors, “a shortage of prototypical features that are most frequently encountered in patients with the disease, features encountered in advanced presentations of the disease or simply features of the disease commonly listed in medical textbooks,” which was developed by Kostopoulou et al. from the systematic review focused on diagnostic errors in primary care,^14^ seems to be comprehensive. Indeed, this definition has been used in other studies.^81,82^ However, this definition has several limitations. First, the definition may be too sensitive to capture the cases with atypical presentations,^82^ which can involve cases not at risk of diagnostic errors. Second, the definition alone does not allow grading or classifying atypical presentations, although there should be a range of atypicality in the cases with atypical presentations (e.g., from slight to quite atypical). Indeed, the Revised Safer Dx Instrument, the standardized tool to detect diagnostic errors through a comprehensive assessment of the diagnostic process, provides the Likert scale to assess disease typicality.^83^ As we showed in this review, some studies also used the Likert scale to measure the grade of atypicality.^16,18,43,46^ Furthermore, a previous study showed several patterns of atypical presentations and suggested that the prevalence of diagnostic errors varies among the grades of atypical presentations.^16^

We propose the PSUC classification as a pragmatic tool for research and diagnostic safety efforts. Any case can be described by the presence or absence of four domains (Primary, Suggestive, Uncommon, and Chameleon), classified into 16 categories. Detailed operational definitions appear in Appendix 2. From the synthesis, we identified four patterns of atypical presentations that were highly associated with diagnostic errors, albeit requiring validation. Beyond conceptual clarity, the PSUC categories may also serve practical scholarly purposes. They offer a common data element that investigators can code across diseases, enable stratified analyses of diagnostic risk, and provide a shared teaching measure for case review, simulation, and audit and feedback programs. Integrating PSUC tagging with tools such as the Revised Safer Dx Instrument may help harmonize measurement across studies. The PSUC framework is intended for qualitative reflection and communication, not numeric scoring. Clinical context and judgement remain paramount, and the PSUC framework should not be used in isolation for decision-making or for quantifying diagnostic difficulty.

## CONCLUSIONS

This scoping review revealed that only a few studies have investigated the association between atypical presentations and diagnostic errors for most acute and chronic diseases. We also highlighted the tremendous heterogeneity of definitions for atypical presentations among the sources of evidence, which may be a barrier to future research and implementation activities to address atypical presentations at high risk of diagnostic errors. The review helped develop a pragmatic approach to classify atypical presentations (the PSUC approach) and identified patterns at higher risk of diagnostic errors. The application of this approach should be tested using real-world patient data.

## Supplementary Information

Below is the link to the electronic supplementary material.

Supplementary Material 1 (DOCX 18.1 KB)
