# Supplementary material for: Atypical Presentations at Risk for Diagnostic Errors in Internal Medicine: A Scoping Review
Source: J Gen Intern Med. 2025 Oct 14;41(7):1937–56. doi: 10.1007/s11606-025-09901-z (PMC13176419; doi:10.1007/s11606-025-09901-z)
Supplement: Supplementary file 1 — Supplementary Material 1 (DOCX 18.1 KB) [file 11606_2025_9901_MOESM1_ESM.docx]

**Appendix 1. MEDLINE (PubMed) Search Strategy**

**Original search (Literature Search Performed: February 5, 2024)**

#1: "Diagnostic Errors"[MeSH Terms] OR "delayed diagnosis"[MeSH Terms] OR "diagnostic error*"[Text Word] OR "misdiagnos*"[Text Word] OR "delayed diagnos*"[Text Word] OR "diagnostic delay*"[Text Word] OR "delay in diagnos*"[Text Word] OR "delays in diagnos*"[Text Word] OR "diagnostic safet*"[Text Word] OR "diagnostic failure*"[Text Word] OR "diagnostic mistake*"[Text Word] OR "inaccurate diagnos*"[Text Word] OR "diagnostic uncertainty"[Text Word] OR "diagnostic challenge*"[Text Word] OR "challenge-in-diagnosis"[Text Word] OR "challenges-in-diagnosis"[Text Word] OR "diagnostic pitfall*"[Text Word] OR "pitfall-in-diagnosis"[Text Word] OR "pitfalls-in-diagnosis"[Text Word] OR "diagnostic blind spot*"[Text Word] OR "diagnostic discrepanc*"[Text Word] OR "wrong diagnos*"[Text Word] OR "missed diagnos*"[Text Word]

#2: ("cognitive error*"[Text Word] OR "cognitive failure*"[Text Word] OR "cognitive bias*"[Text Word] OR ("cognition"[MeSH Terms] AND "bias"[MeSH Terms]) OR ("anchoring"[Text Word] AND "bias*"[Text Word]) OR "ascertainment bias*"[Text Word] OR "availability bias*"[Text Word] OR "base-rate-neglect"[Text Word] OR "commission bias*"[Text Word] OR "confirmation bias*"[Text Word] OR "diagnostic momentum"[Text Word] OR "framing effect*"[Text Word] OR "fundamental attribution error*"[Text Word] OR "gambler's fallacy"[Text Word] OR "gamblers fallacy"[Text Word] OR "gender bias*"[Text Word] OR "hindsight bias*"[Text Word] OR "illusory correlation*"[Text Word] OR "status quo bias*"[Text Word] OR "wallpaper phenomenon*"[Text Word] OR "omission bias*"[Text Word] OR "order effect*"[Text Word] OR "outcome bias*"[Text Word] OR "overconfidence bias*"[Text Word] OR "value bias*"[Text Word] OR "playing the odd*"[Text Word] OR "posterior probability error*"[Text Word] OR "premature closure*"[Text Word] OR "sunk cost*"[Text Word] OR "unpacking principle*"[Text Word] OR "affective bias*"[Text Word] OR "zebra retreat*"[Text Word] OR "ambiguity effect*"[Text Word] OR "representativeness bias*"[Text Word] OR "unconscious bias*"[Text Word] OR "cognitive dispositions to respond*"[Text Word] OR "heuristic*"[Text Word] OR "heuristics"[MeSH Terms] OR "unconscious bias*"[Text Word] OR "missed opportunit*"[Text Word]) AND ("clinical decision making"[MeSH Terms] OR "clinical decision*"[Text Word] OR "clinical reasoning"[Text Word] OR "Decision Making"[Text Word] OR "Decision Making"[MeSH Terms] OR "diagnos*"[Text Word] OR "common disease*"[Text Word] OR "common symptom*"[Text Word] OR "typical disease*"[Text Word] OR "typical symptom*"[Text Word])

#3: #1 OR #2

#4: "atypical"[Text Word] OR "uncommon"[Text Word] OR "abnormal"[Text Word] OR "unusual"[Text Word] OR "nonclassical*"[Text Word] OR "non classical"[Text Word] OR "rare"[Text Word]

#5: #3 AND #4

#6 "internal medicine"[Text Word] OR "general internal medicine"[Text Word] OR "general medicine"[Text Word] OR "hospital medicine"[Text Word] OR "internal medicine"[MeSH Terms] OR "hospital medicine"[MeSH Terms] OR "hospitalists"[MeSH Terms] OR "internist*"[Text Word] OR "hospitalist*"[Text Word] OR "physician*"[Text Word] OR "physicians"[MeSH Terms] OR "nurse practitioner*"[Text Word] OR "Nurse Practitioners"[MeSH Terms]

#7: #5 AND #6

#8: 0001/01/01:2023/12/31[Date - Create]

#9: #7 AND #8

#10: "case reports"[Publication Type] OR "letter"[Publication Type] OR "comment"[Publication Type] OR "case*"[Title]

#11: #9 NOT #10

**Updated search (Literature Search Performed: August 18, 2025)**

#1: "Diagnostic Errors"[MeSH Terms] OR "delayed diagnosis"[MeSH Terms] OR "diagnostic error*"[Text Word] OR "misdiagnos*"[Text Word] OR "delayed diagnos*"[Text Word] OR "diagnostic delay*"[Text Word] OR "delay in diagnos*"[Text Word] OR "delays in diagnos*"[Text Word] OR "diagnostic safet*"[Text Word] OR "diagnostic failure*"[Text Word] OR "diagnostic mistake*"[Text Word] OR "inaccurate diagnos*"[Text Word] OR "diagnostic uncertainty"[Text Word] OR "diagnostic challenge*"[Text Word] OR "challenge-in-diagnosis"[Text Word] OR "challenges-in-diagnosis"[Text Word] OR "diagnostic pitfall*"[Text Word] OR "pitfall-in-diagnosis"[Text Word] OR "pitfalls-in-diagnosis"[Text Word] OR "diagnostic blind spot*"[Text Word] OR "diagnostic discrepanc*"[Text Word] OR "wrong diagnos*"[Text Word] OR "missed diagnos*"[Text Word]

#2: ("cognitive error*"[Text Word] OR "cognitive failure*"[Text Word] OR "cognitive bias*"[Text Word] OR ("cognition"[MeSH Terms] AND "bias"[MeSH Terms]) OR ("anchoring"[Text Word] AND "bias*"[Text Word]) OR "ascertainment bias*"[Text Word] OR "availability bias*"[Text Word] OR "base-rate-neglect"[Text Word] OR "commission bias*"[Text Word] OR "confirmation bias*"[Text Word] OR "diagnostic momentum"[Text Word] OR "framing effect*"[Text Word] OR "fundamental attribution error*"[Text Word] OR "gambler's fallacy"[Text Word] OR "gamblers fallacy"[Text Word] OR "gender bias*"[Text Word] OR "hindsight bias*"[Text Word] OR "illusory correlation*"[Text Word] OR "status quo bias*"[Text Word] OR "wallpaper phenomenon*"[Text Word] OR "omission bias*"[Text Word] OR "order effect*"[Text Word] OR "outcome bias*"[Text Word] OR "overconfidence bias*"[Text Word] OR "value bias*"[Text Word] OR "playing the odd*"[Text Word] OR "posterior probability error*"[Text Word] OR "premature closure*"[Text Word] OR "sunk cost*"[Text Word] OR "unpacking principle*"[Text Word] OR "affective bias*"[Text Word] OR "zebra retreat*"[Text Word] OR "ambiguity effect*"[Text Word] OR "representativeness bias*"[Text Word] OR "unconscious bias*"[Text Word] OR "cognitive dispositions to respond*"[Text Word] OR "heuristic*"[Text Word] OR "heuristics"[MeSH Terms] OR "unconscious bias*"[Text Word] OR "missed opportunit*"[Text Word]) AND ("clinical decision making"[MeSH Terms] OR "clinical decision*"[Text Word] OR "clinical reasoning"[Text Word] OR "Decision Making"[Text Word] OR "Decision Making"[MeSH Terms] OR "diagnos*"[Text Word] OR "common disease*"[Text Word] OR "common symptom*"[Text Word] OR "typical disease*"[Text Word] OR "typical symptom*"[Text Word])

#3: #1 OR #2

#4: "atypical"[Text Word] OR "uncommon"[Text Word] OR "abnormal"[Text Word] OR "unusual"[Text Word] OR "nonclassical*"[Text Word] OR "non classical"[Text Word] OR "rare"[Text Word]

#5: #3 AND #4

#6 "internal medicine"[Text Word] OR "general internal medicine"[Text Word] OR "general medicine"[Text Word] OR "hospital medicine"[Text Word] OR "internal medicine"[MeSH Terms] OR "hospital medicine"[MeSH Terms] OR "hospitalists"[MeSH Terms] OR "internist*"[Text Word] OR "hospitalist*"[Text Word] OR "physician*"[Text Word] OR "physicians"[MeSH Terms] OR "nurse practitioner*"[Text Word] OR "Nurse Practitioners"[MeSH Terms]

#7: #5 AND #6

#8: 2024/01/01:2025/07/31[Date - Create]

#9: #7 AND #8

#10: "case reports"[Publication Type] OR "letter"[Publication Type] OR "comment"[Publication Type] OR "case*"[Title]

#11: #9 NOT #10

**Appendix 2. Operational definitions of the PSUC framework and examples**

When any features listed in the major diagnostic criteria or the definition of the target disease are present, the primary feature is considered positive. Suggestive features can be positive when there are features listed in the minor diagnostic criteria, features have high positive likelihood ratio, features known as one of the red flags such as unintentional weight loss for any malignancy, features known as the high risk profiles such as decompensated cirrhosis for hepatocellular carcinoma and combinations of risk factors such as four Fs for gallstones, or high-yield diagnostic features. Uncommon features include infrequent features, uncommon sites of lesions for the target disease such as cutaneous lesion of tuberculosis, metastasis of cancer to uncommon sites such as liver metastasis of early stage of lung cancer or renal cancer, or uncommon order of the development of symptoms, signs, or findings such as fever as the initial symptom in acute appendicitis. Chameleon features are not the primary features of the target disease but are the primary features of another disease, and suggestive and uncommon features can also be chameleon features at the same time.

The PSUC framework can be flexibly applied to the contextuality of clinical presentations, and the same features can be used in other classification cases on a case-by-case basis. We simulate an example case of an older adult with chronic obstructive pulmonary disease (COPD) who presents with isolated acute shortness of breath (no chest pain) and has an elevated troponin and nonspecific electrocardiogram findings. The final diagnosis is acute coronary syndrome. In this case, we consider an elevated troponin level as the primary feature and acute shortness of breath as a suggestive feature, with no uncommon or chameleon features. While shortness of breath is a less common symptom of acute coronary syndrome, it should be considered a sign of acute coronary syndrome in older adults. Since primary features exist (elevated troponin), shortness of breath cannot usually be a chameleon feature. In this case (relatively typical presentation), when the case is initially diagnosed as COPD exacerbation (wrong diagnosis), patient, contextual (environmental), and system-level factors could be the primary reasons for diagnostic errors.

On the other hand, if troponin level is normal in the case, the case can be classified as both the suggestive and chameleon features present and both the primary and uncommon features absent, because isolated acute shortness of breath in older patients with COPD is the primary feature of COPD exacerbation (chameleon feature) even though acute shortness of breath is still a suggestive feature of acute coronary syndrome. In this case, higher situational awareness and sufficient knowledge with experience for atypical presentations of acute coronary syndrome and thorough consideration of differential diagnosis are required for accurate diagnosis of acute coronary syndrome. Therefore, to reduce diagnostic errors in this case (which have relatively atypical presentations), individual or team-level education, simulation, and reflection on similar cases may be optimal. Moreover, even in the same final diagnosis of acute coronary syndrome, if the case is a young woman with asthma who presents with isolated acute shortness of breath (no chest pain) and has normal troponin and nonspecific electrocardiogram findings, we consider shortness of breath as an uncommon feature as well as a chameleon feature. Primary and suggestive features are absent. Whether such a case is regarded as a diagnostic error or not can be debatable, but improving diagnosis in this type of presentation (quite atypical presentations) is also required. As shown above, the PSUC classification may facilitate consideration of the major contributing factor to diagnostic errors and the type of interventions required in each case. For practical purposes, each feature is coded as “1” if present and “0” if absent (e.g., P1 S0 U0 C1).
